# TNF-driven cell fate: till HACE do us part

**DOI:** 10.18632/oncotarget.10168

**Published:** 2016-06-19

**Authors:** Luigi Tortola, Roberto Nitsch, Josef M. Penninger

**Affiliations:** IMBA, Institute of Molecular Biotechnology of the Austrian Academy of Sciences, Vienna, Austria

**Keywords:** HACE1, TNF, NFkB, apoptosis, necroptosis

Tissue homeostasis is a fine balance between cell survival, proliferation, differentiation and cell death. While tissue integrity needs to be maintained by ensuring cell survival even in adverse conditions, the replacement of damaged or compromised cells is just as important to preserve functionality of the tissue. This equilibrium between cell survival and death is crucial for life and plays a decisive role in development and in the control of inflammation and tumorigenesis. The different pathways regulating programmed cell death serve this purpose. Apoptosis was the first described type of programmed cell death. Both intrinsic and extrinsic cues can lead to the activation of caspases that in turn initiate an effector cascade culminating in cell death. Apoptosis can be viewed as a “silent implosion”: the dying cell collapses but its content is not released into the surrounding environment, which prevents the onset of inflammatory responses that might be detrimental for tissue integrity [1]. However, other pathways of programmed cell death exist. Among them, RIPK1/RIPK3/MKLK-dependent programmed necrosis, or necroptosis, represents a type of cell death that, unlike apoptosis, leads to the release of cell content and of so-called “danger signals”, thus promoting immune cell activation and inflammation [2].

Interestingly, in some cases cell survival, apoptosis and necroptosis can be triggered by the very same cellsurface receptors. This is the case for the tumor necrosis factor (TNF)-receptor 1 (TNFR1). This paradigmatic signaling pathway was shown to induce dramatically different responses depending on the context of activation. While TNF can trigger the activation of the transcription factor NFκB and thereby expression of pro-survival factors, in conditions in which this pathway is blocked TNF can drive the induction of apoptosis or necroptosis instead.

Cell fate decisions regulating survival, apoptosis and necroptosis are critical for the maintenance of homeostasis. Imbalances in this equation can lead to the development of tumors and immune pathologies. For instance deregulated induction of necroptosis was shown to be associated with the onset of intestinal inflammation [3]. Unraveling the network of regulators controlling such life and death cell fate decisions therefore holds the key to a better understanding of the mechanisms underlying development of inflammatory pathologies and cancer and to the development of new drugs.

In this respect, we recently identified the key role of the HECT-domain and ankyrin-repeat containing E3 ligase 1 (HACE1) in determining the fate downstream of TNFR1 [4]. HACE1 was first described in the context of sporadic Wilms’ tumor. In this study and others that followed, HACE1 was shown to act as a tumor suppressor: downregulation of this ubiquitin ligase was observed in many types of human tumors and we showed that genetic inactivation of *Hace1* causes an increased predisposition to the development of cancer [5].

**Figure 1:**
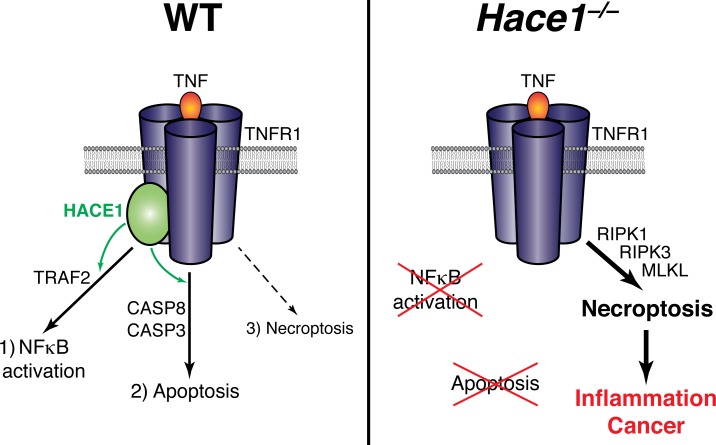
HACE1 controls cell fate in response to TNFR1 ligation Upon stimulation with TNF, HACE1 mediates the induction of NFκB activation and apoptosis, thereby maintaining a balance among the possible cell fates downstream of TNFR1 (left panel). In the absence of HACE1, NFκB activation and apoptosis induction downstream of TNFR1 are impaired and cells are predisposed to necroptotic death, which promotes intestinal inflammation and carcinogenesis (right panel).

Aiming to discern the molecular basis for the tumor suppressor function of HACE1, we set out to identify its ubiquitylation substrates. Intriguingly, among the targets of HACE1 we discovered several proteins that were previously implicated in TNF and NFκB signaling. Indeed, signaling downstream of TNF receptor was strongly affected by the presence or absence of HACE1: NFκB activation was largely abrogated in *Hace1*- deficient cells stimulated with TNF. This did not, however, result in a higher susceptibility to apoptotic cells death. On the contrary, *Hace1−/−* cells showed impaired caspase activation and defective induction of apoptosis when treated with TNF in the presence of inhibitors of the NFκB-mediated survival pathway or Smac mimetics. However, while these two key pathways downstream of TNFR1 were impaired, TNF-driven RIPK1/RIPK3/MLKL-dependent necroptosis was perfectly functional in the absence of HACE1. This data highlights HACE1 as a gatekeeper of TNF-driven cell fate (Figure 1). In its absence, TNF-driven NFκB activation and apoptosis are abrogated and cells get predisposed to undergo necroptotic cell death.

These alterations in TNF-mediated cell fate in the absence of HACE1 have functional corollaries *in vivo*. *Hace1−/−* animals were protected from TNF/DGalactosamine- mediated liver damage and death. Also, we found *Hace1−/−* animals to be predisposed to the development of intestinal inflammation and of inflammation-driven colon cancer. Importantly, this phenotype could be reverted by genetic inactivation of *Tnfr1* and *Ripk3*, indicating that increased colonic inflammation and carcinogenesis in the absence of HACE1 depend on deregulated TNF-driven necroptosis (Figure 1).

Which of the substrates of HACE1 is responsible for the regulation of TNFR1 signaling? We observed that HACE1 directly mediates K63-linked ubiquitylation of TRAF2, which was previously shown to induce NFκB activation and inhibit necroptosis downstream of TNFR1 [6, 7]. While it is tempting to speculate that this particular modification of TRAF2 is responsible for the alterations in TNF-driven cell fate in the absence of HACE1, future work will have to focus on defining how TRAF2 and other candidate ubiquitylation targets of HACE1 interfere with signaling downstream of TNFR1.
